# Estrogen-producing endometrioid adenocarcinoma resembling sex cord-stromal tumor of the ovary: a review of four postmenopausal cases

**DOI:** 10.1186/1746-1596-7-164

**Published:** 2012-11-28

**Authors:** Tomomi Katoh, Masanori Yasuda, Kosei Hasegawa, Eito Kozawa, Jun-ichi Maniwa, Hironobu Sasano

**Affiliations:** 1 Departments of Pathology, Saitama Medical University International Medical Center, 1397-1Yamane, Hidaka, Saitama 350-1298, Japan; 2 Departments of Gynecological Oncology, Saitama Medical University International Medical Center, 1397-1Yamane, Hidaka, Saitam, 350-1298, Japan; 3 Departments of Radiology, Saitama Medical University International Medical Center, 1397-1Yamane, Hidaka, Saitama 350-1298, Japan; 4 Division of Pathology, Kumagaya General Hospital, Kumagaya, Saitama, Japan; 5 Department of Pathology, Tohoku University Hospital, Sendai, Miyagi, Japan

**Keywords:** Ovary, Postmenopausal, Estrogen (E2) overproduction, Endometrioid adenocarcinoma, Resembling sex cord-stromal tumor

## Abstract

**Abstract:**

The 4 present cases with endometrioid adenocarcinoma (EMA) of the ovary were characterized by estrogen overproduction and resemblance to sex cord-stromal tumor (SCST). The patients were all postmenopausal, at ages ranging from 60 to 79 years (av. 67.5), who complained of abdominal discomfort or distention and also atypical genital bleeding. Cytologically, maturation of the cervicovaginal squamous epithelium and active endometrial proliferation were detected. The serum estrogen (estradiol, E2) value was preoperatively found to be elevated, ranging from 48.7 to 83.0 pg/mL (av. 58.4). In contrast, follicle stimulating hormone was suppressed to below the normal value. MR imaging diagnoses included SCSTs such as granulosa cell tumor or thecoma for 3 cases because of predominantly solid growth, and epithelial malignancy for one case because of cystic and solid structure. Grossly, the solid part of 3 cases was homogeneously yellow in color. Histologically, varying amounts of tumor components were arranged in solid nests, hollow tubules, cord-like strands and cribriform-like nests in addition to the conventional EMA histology.

In summary, postmenopausal ovarian solid tumors with the estrogenic manifestations tend to be preoperatively diagnosed as SCST. Due to this, in the histological diagnosis, this variant of ovarian EMA may be challenging and misdiagnosed as SCST because of its wide range in morphology.

**Virtual slides:**

http://www.diagnosticpathology.diagnomx.eu/vs/6096841358065394

## Introduction

Endometrioid adenocarcinoma (EMA) may occur as a unique variant, irrespective being of ovarian origin or uterine endometrial origin [1]. The patients with ovarian tumors often complain variable and unusual symptoms [2]. Some postmenopausal patients with ovarian tumor present with atypical genital bleeding. In addition to this symptom, when endometrial thickening is detected on the imaging examination, the possibility of estrogen overproduction by the ovarian tumor can be raised. Cervicovaginal cytology also shows an increased maturation of squamous epithelium due to estrogenic effects [3]. As one of the most representative ovarian tumors with estrogen overproduction in postmenopausal women, adult granulose cell tumor is routinely encountered. Theco-fibromatous tumor and Brenner tumor arising in postmenopausal women are also known as having a potential to produce estrogen more than the normal range.

Since 3 to 4 decades ago, postmenopausal epithelial ovarian tumors have been found to overproduce estrogen with considerable frequency [4,5]. According to the certain reports, mucinous tumor is most frequently characterized by estrogen overproduction [6-8]. In the other histological types such as serous, endometrioid and clear cell tumors, however, the recognition for their potential to overproduce estrogen seems to be less generalized, not only for pathologists but also for gynecologists.

In our institution, endocrinological examination including the serum value of estrogen (E2) and follicle stimulating hormone (FSH) was performed for solid ovarian tumors arising in postmenopausal women, especially when they present with atypical genital bleeding, endometrial thickening and/or an increased maturation of squamous epithelium. As a result, 6 cases with ovarian EMA having E2 overproduction were encountered during the last 5 years in our institution. Among them, 4 cases were diagnosed as EMA resembling sex cord-stromal tumor (SCST). In addition to EMA, a case with serous adenocarcinoma and 3 cases with clear cell adenocarcinoma which showed estrogen overproduction were also experienced. Based on the histological observation alone, it is less easy to determine whether or not the ovarian epithelial tumors may overproduce estrogen. As a tumor marker in the ovarian tumors, estrogen may have a clinically significant implication [5-7].

## Case presentation

**Figure 1:**
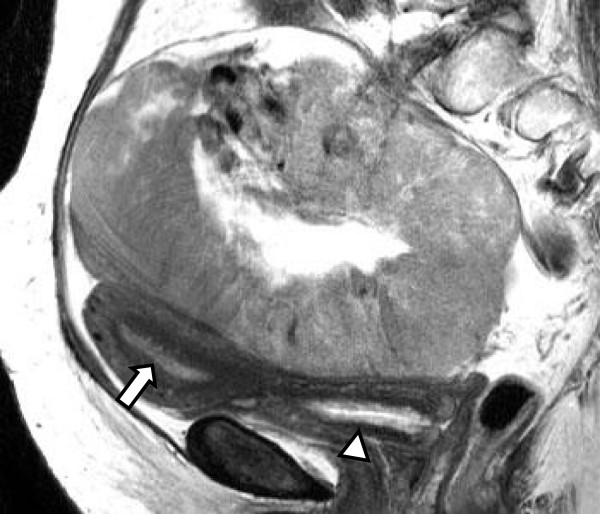
T2-enhanced MR imaging demonstrates a solid mass measuring 14 cm in the great diameter in the pelvic cavity. The uterus is found to have a thickened endometrium (arrow) and endocervical glands (arrow head) (Case 3).

The patients were all postmenopausal, aged between 60 and 79 years (av. 67.5). Their chief complaints were atypical genital bleeding and abdominal distention or discomfort. Preoperative endocrinological abnormalities included elevation of serum E2 and suppression of FSH (Table 1). Cytologically, maturation of squamous epithelium increased on the cervicovaginal smear in 3 cases. Active proliferative condition of the endometrium was detected cytologically in the endometrial smear and histologically in the endometrial biopsy in 3 cases. MR imaging displayed that the ovarian tumors were predominantly solid (Figure 1) except for Case 4, in which the tumor was cystic and solid. Uterine enlargement was demonstrated in Cases 1, 3 and 4, and the endometrium was thickened in Cases 2 and 3. Preoperatively, the differential diagnostic considerations were granulosa cell tumor, thecoma or fibroma, and Brenner tumor. All patients underwent total hysterectomy and bilateral adnexectomy with or without omentectomy. No lymph node dissection was performed for any of the patients. Postoperative chemotherapy with administration of Paclitaxel and Carboplatin was performed for Case 4. All of the patients have taken an uneventful clinical course after surgery or chemotherapy during a period ranging from 7 to 48 months (av. 33). The serum levels of E2 and FSH postoperatively returned to their normal ranges.

**Table 1:** Clinicopathological presentations

	**case 1**	**case 2**	**case 3**	**case 4**
age	60	61	70	79
menopausal age	52	49	52	53
complaint	atypical genital bleeding	atypical genital bleeding	abdominal distension	no symptom
^a^ E2 (pg/ml)	52.0	48.7	83.0	50.0
^a^ FSH (mIU/ml)	4.8	8.4	6.9	23.4
preoperative diagnosis	GCT or thecoma	thecoma	GCT or thecoma	thecoma, fibroma or Brenner tumor
treatment	TAH, BSO	TAH, BSO	TAH, BSO, OMT	TAH, BSO, OMT, TCx6
FIGO stage	IA	IA	IA	IC
follow-up	NED 48 months	NED 38 months	NED 7 months	NED 39 months
side	left	left	right	left
size (cm)	9x9	10x9	16x7	10x8
gross	solid/yellow	solid/yellow	solid/yellow	solid and cystic/gray-white
cervicovaginal smear (MI)	not available	0/10/90	5/30/65	5/20/75
endometrial smear	not available	active	active	not performed
SCST components	20% (SCT)	50% (SCT>GCT)	80% (GCT>SCT)	50% (SCT)
other components	-	EMAF	-	EMAF and MCA
endometriosis	-	+	-	-
functioning stroma	+++	+	+++	+

^a^ pre-operative.

FSH: follicle stimulating hormone, TAH: total abdominal hysterectomy, BSO: bilateral salpingo-oophorectomy.

OMT: omentectomy, TC: paclitaxel+ carboplatin, NED: no evidence of desease.

SCST: sex-cord stromal tumor, SCT: Sertori cell tumor, GCT: granulosa cell tumor.

EMAF: endometrioid adenofibroma, MCA: mucinous cystadenofibroma.

## Pathological findings

### Gross

**Figure 2:**
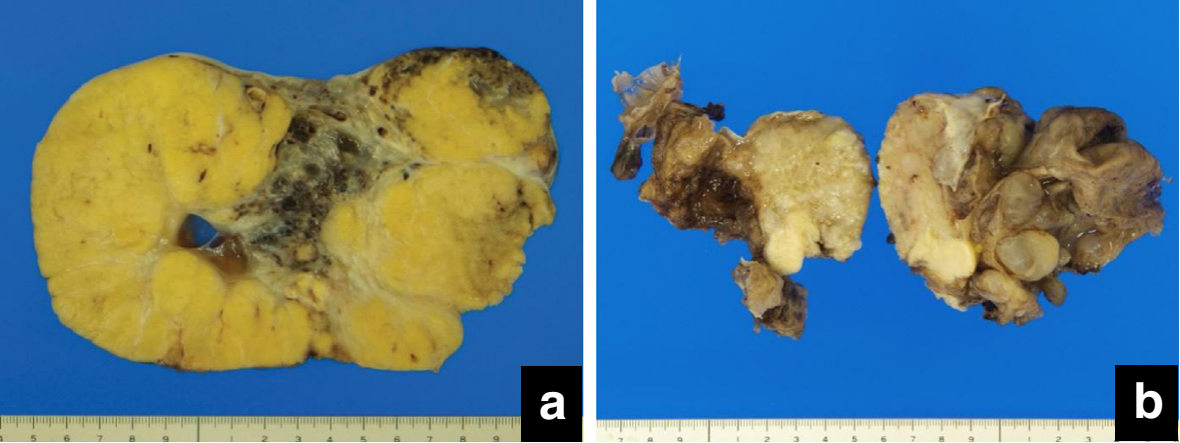
Cut surface view a (Case 3): The tumor is homogeneously yellow in color and irregularly lobulated, but focally hemorrhagic and necrotic. b (Case 4): The majority of the tumor is microcystic and filled with mucous substances, and focally contains a solid part (arrow).

The ovarian tumors were all unilateral, and their size measured from 9 to 16 cm in the great diameter (Table 1). The cut surface of Cases 1, 2 and 3 was predominantly solid and homogenously yellow, with focal necrosis or hemorrhage (Figure 2a), but that of Case 4 showed in the major part microcystic components and a focally solid part (Figure 2b).

### Histology

**Figure 3:**
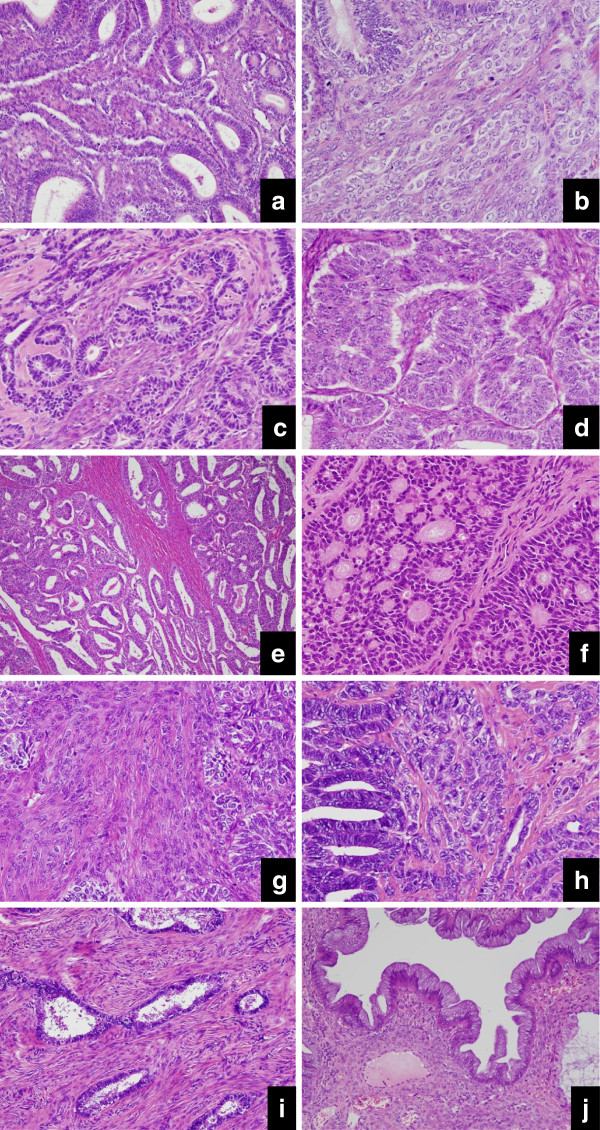
Microscopic findings a (Case 1): Cord-like tubules, mimicking Sertoli cell tumor, are showing a transition with endometrioid components. b (Case 1): Abundant stromal cells, possessing round nuclei and pale cytoplasm, are characterized by luteinization. c (Case 2): Small-sized tubular nests, mimicking Sertoli cell tumor, are found to be fused. d (Case 2): Solid nests with no tubular structure are showing the appearance of granulosa cell tumor. e (Case 3): This area is composed of conventional EMA. f (Case 3): Large-sized tubular nests with cribriform-like structure mimic the Call-Exner body. g (Case 3): Abundant stroma is rather condensed, but lacks typical luteinization. h (Case 4): Small-sized nests or strands resemble the features of a Sertoli cell tumor. i (Case 4): A part of adenofibromatous endometrioid tumor is shown. j (Case 4): The major part of the tumor is composed of mucinous cystadenoma.

The ovarian tumors showed an admixture of conventional EMA and components resembling SCST with/without transition between them. The components resembling SCST accounted for 20 to 80% of the entire tumor (Table 1). The histological findings of each tumor were as follows: Case 1 showed elongated and cord-like or thin tubules, resembling Sertoli cell tumor, in continuity with endometrioid components (Figure 3a) and abundant stromal cells with round nuclei and pale cytoplasm, which were characterized by luteinization (Figure 3b); Case 2 showed small-sized, fused tubular nests resembling Sertoli cell tumor, accompanied by abundant stroma (Figure 3c), and solid nests resembling granulosa cell tumor (Figure 3d). Bland glands resembling endometrial glands, which were regarded as endometriosis, focally existed; Case 3 showed tubular nests of usual EMA (Figure 3e), cribriform-like nests simulating Call-Exner bodies (Figure 3f), and condensed stroma intervening among the solid nests; Case 4 showed tubular nests and small-sized nests or strands (Figure 3h), benign counterparts of adenofibromatous endometrioid tumor (Figure 3i) and mucinous cystadenoma (Figure 3j). The presence of functioning stroma was indicated in Cases 1 (Figure 3) and 3 (Figure 3g). However, in Cases 2 and 4, the stroma was less cellular or hyalinized, lacking typical luteinization.

The endocervical glands of the hysterectomy specimens were more activated in the secretion compared to those in postmenopausal patients with non-estrogen producing ovarian tumor. The endometrial glands seemed to be functioning irrespective of the patients’ postmenopausal status. These endocervical and endometrial findings were in concordance with the cytological findings of the cervicovaginal smear and the endometrial smear, respectively.

The FIGO stage was determined at IA for Cases 1, 2, and 3, and IC for Case 4.

### Immunohistochemistry

**Figure 4:**
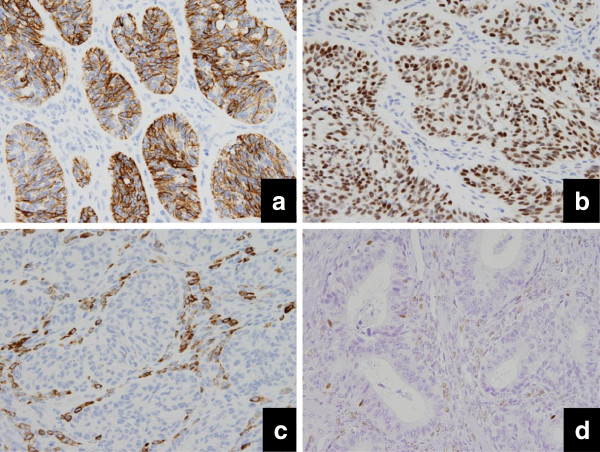
**Immunohistochemically, the carcinoma cells are clearly positive for CK7 (a), ER (b), while many stromal cells show positive reaction for inhibin-α (c) (Case 3). Expression of steroid factor-1 (SF-1), which is a transcription factor alternatively termed adrenal 4-binding protein (Ad4BP) to regulate expression of all steroidogenic enzymes **[9]**, is predominant in the stromal cells but minimal in epithelial cells (Case 1).**

The expressions of CK7 (clone: OV-TL 12/30, 1:50, Dako, Glostrup, Denmark), epithelial membrane antigen (clone: E29, 1:50, Dako, Glostrup, Denmark), estrogen receptor (ER, clone: SP-1, 1:1, Roche, AZ, USA), inhibin-α (clone: R1, 1:50, Serotec, Oxford, UK), and steroidogenic factor-1 (SF-1) [9-11] were examined. As a result, the positive immunoreactions of CK7 (Figure 4a), epithelial membrane antigen and ER (Figure 4b) were clearly and massively observed in EMA resembling SCST as well as in conventional EMA. Inhibin-α (Figure 4c) was positively observed in the stromal cells but negatively observed in the epithelial tumor cells. SF-1 (Figure 4d) expression was abundant in the stromal cells but was also present to a lesser degree in the epithelial tumor cells.

## Discussion

Ovarian EMA has been known for approximately 30 years to have a histological variant which is characterized by a resemblance to SCST [12,13], but the precise frequency of EMA resembling SCST remains to be clarified. According to Young et al., 8 of 13 cases were initially misdiagnosed as SCST [12]. A lack of accurate recognition about this variant may yield a misinterpretation which results in the misdiagnosis as SCST instead of EMA. Especially, at the intraoperative diagnosis, both the macroscopic and microscopic findings may mislead pathologists to raise the possibility of SCST. Although our experience is limited, the 4 present cases were encountered during the last 5 years since the opening of our institution, accounting for approximately 10% of the total ovarian EMAs. In fact, Case 1 (Figure 3a, b) was initially diagnosed by the referring pathologist as Sertoli-Leydig cell tumor with estrogen overproduction, mainly because of his misinterpreting the luteinized stromal cells as Leydig cells. One of the critical differential points between Sertoli-Leydig cell tumor and EMA is the occurrence age: 28 years for the former and over 55 years for the latter [12]. But, interestingly, it should be noted that some Sertoli-Leydig cell tumors contain areas which resemble endometrioid tumor [14]. As for Case 2 (Figure 3c, d), at the intraoperative diagnosis, the possible diagnosis of Sertoli cell tumor was made by the referring pathologist. In this case, the presence of granulosa cell tumor-like components also may have become a key to misdiagnosis.

It has not been pathologically defined as to what part of the components resembling SCST is supposed to be contained for the diagnosis of this variant. Reportedly, the proportions of components resembling SCST in the entire tumor vary considerably, ranging from 30 to 100% [15]. In our review, too, the proportions ranged from 20 to 80%. Except for EMA which is almost entirely occupied by the components resembling SCST, as in Case 3 (Figure 3e, f, g), a combination with a variable amount of conventional EMA would favor the diagnosis of EMA resembling SCST. Though relatively rare, a coexistence of cribriform-like nests and solid nests may make it difficult to lead to the accurate diagnosis of a variant of EMA. In addition, the histologically reliable hallmarks of EMA are as follows: squamous differentiation of the tumor cells, complication of endometriosis within the tumor or in the background, and transition between adenofibromatous components of endometrioid type and carcinoma [12]. In Case 2, endometriosis was speculated to be associated with tumor development. In Case 4 (Figure 3h, i, j), a transition with the benign component of adenofibromatous endometrioid tumor was considered to be strongly supportive of the diagnosis of a variant of EMA. But, it should be noted that mucinous cystadenoma may coexist not only in other epithelial tumors but also in SCSTs. Interestingly, in this case, the stromal cells were found to be more cellular in the vicinity of mucinous cystadenoma rather than within the endometrioid benign and malignant tumors.

When histologically challenging cases are encountered, certain immunohistochemical markers are thought to contribute greatly to making the diagnosis definite. The epithelial nature of EMA, in spite of the resemblance to SCST, could be determined by positive reactions for CK7 and epithelial membrane antigen [15-17]. Many EMAs are positive for ER [15] but inconsistently negative for inhibin-α [15,17]. In the cases with pure SCST, inhibin-α is frequently expressed, while these epithelial markers are less frequently or weakly expressed if at all [15-17], and ER expression is usually negative [18].

The first descriptions about the ovarian functioning stroma go far back to the 1950s [19]. This stromal change has been considered to be closely associated with clinical, biochemical or pathological evidence of endocrine function [4-7]. Several investigators demonstrated that many postmenopausal women with a variable epithelial ovarian tumor would be provided with a potential to produce more sex steroid hormone represented by estrogen than normal postmenopausal women [6-8,20,21]. The frequency of estrogen overproduction is reported to be over 70% by serological examinations [7,8], and 47% [4] or 50% [5] by urine analyses. According to some reports, steroidogenic cells are observed more frequently in mucinous tumors [6,8,20], and predominantly in postmenopausal women (80%) [21]. However, the difference in the incidence of estrogen overproduction among the variable types of ovarian tumors remains controversial [22]. Concerning the difference among benign tumor, borderline malignancy, and malignant tumor, the previous reports do not seem to present confirmative data because of their statistically small scale of examined cases as well as their deviation in histological types [6-8,20,21].

In the report by Young et al., the association between EMA resembling SCST and estrogenic manifestation or presence of steroidogenic stroma was described as follows: 4 of 13 cases had the stroma consistent with that of luteinized stromal cells, but the evidence of possible estrogen overproduction was noted in only one case [12]. There is another description that theca-like stroma and foci of vacuolated lutein-like cells were noted in one of 4 cases with EMA [13]. To the best of our knowledge, with regard to postmenopausal EMA with estrogen overproduction which was confirmed by serological examination, there have been only 4 reported cases [11,23-25]. These 4 cases included one case with EMA resembling SCST, and the remaining 3 cases showed conventional EMA. Based on our limited experience, it is speculated that EMA resembling SCST may have more estrogenic manifestations due to the presence of steroidogenic stroma. Focusing on the source of estrogen overproduction and its mechanism, in terms of the morpho-functional correlation, immunohistochemical and molecular-based analyses have been performed [9-11,23,26,27]. Interestingly, Sasano et al. mentioned that SF-1 and steroidogenic enzymes are expressed in not only stromal cells but also epithelial tumor cells [10]. However, the association between clinical hormonal abnormalities and presence or absence of luteinized stromal cells in ovarian tumor, including the relationship with the mechanism of extragonadal aromatization, has not been fully established yet [9].

In summary, EMA resembling SCST should be definitely differentiated from SCST from the clinical and pathological aspects. In order to clarify the significance of estrogen as a tumor marker for the ovarian tumor and its utility in the management of the patient, more precise clinical and pathological data must be gathered. Furthermore, the molecular-based mechanism with estrogen overproduction needs to be explored.

## Competing interests

The authors declare that they have no competing interests.

## Authors’ contributions

TK wrote the manuscript in the most part. MY conducted how to report the present cases. KH analyzed the clinical information as gynecological oncologist. EK took part in this review as an expert of imaging diagnosis. JM supported immunohistochemistry. HS carried out immunohistochemistry. All authors read and approved the final manuscript.
